# Beyond a Game: A Narrative Review of Psychopathic Traits in Sporting Environments

**DOI:** 10.3390/sports11110228

**Published:** 2023-11-15

**Authors:** Jill Colangelo, Alexander Smith, Anna Buadze, Michael Liebrenz

**Affiliations:** 1 Department of Forensic Psychiatry, University of Bern, 3012 Bern, Switzerland; alexander.smith@unibe.ch (A.S.); michael.liebrenz@unibe.ch (M.L.); 2 Department of Psychiatry, Psychotherapy and Psychosomatics, Psychiatric Hospital, University of Zurich, 8091 Zurich, Switzerland; ana.buadze@pukzh.ch

**Keywords:** psychopathy, dark triad, athletes, violence, forensic psychiatry, sport, sports psychiatry

## Abstract

There has been a growing interest around the broader effects of psychopathic traits, particularly in relation to deviant behaviors and the notion of so-called “successful psychopathy”. As significant sociocultural touchstones, sporting events are often characterized by competitiveness and a sense of prestige. However, there has been limited attention towards psychopathic traits across recreational, amateur, and elite sports. Accordingly, we conducted a narrative review synthesizing primary observations on this topic, searching keywords in Scopus, APA PsychNet, and PubMed. Twenty-four academic papers were included in our results, which we thematized around demographic groups, namely: athletes and sport-adjacent non-athletes (i.e., coaches and spectators). Based on empirical findings from the reviewed papers, psychopathic traits could have medicolegal and forensic implications in relation to substance use, aggression, and violence. These could intersect with wider issues around doping, cheating, foul play, and have adverse outcomes for fellow participants, team dynamics, and spectators. Interestingly, our review also indicates that psychopathic traits may have correlations with determination and achievement in sport, echoing developing ideas around “successful psychopathy” in other domains. As such, increased awareness from all stakeholders and further multidisciplinary exchanges are vital to better understand the effects of psychopathic traits in sporting frameworks and their wider consequences.

## 1. Introduction

Influencing behaviors that are antipathetic to social norms [1], psychopathy has been considered to be the most insidious and harmful of the Dark Triad (DT) traits of narcissism, Machiavellianism, and psychopathy [2]. Specifically, psychopathic traits can entail a lack of remorse and empathy in interpersonal relationships, impulsive actions without consideration for safety, and a propensity for antisocial or thrill-seeking behaviors [3,4]. Individuals exhibiting psychopathic traits may be more likely to act in manipulative, grandiose, and dominant ways [4,5]. This can result in the contravention of commonly accepted societal conventions and deviant behaviors [1]. Psychopathic traits can emerge in early life and may be shaped by genetic and social factors [1,4].

Though each component of the DT model (i.e., narcissism, Machiavellianism, and psychopathy) share certain features and correlations with one another, they may encourage different behaviors contingent on individual personality factors [6]. Moreover, scholarly discussion of psychopathy has often been intertwined with the DT construct and with DT psychometric instruments frequently adopted to determine trait status [7,8].

Nonetheless, it should be noted that current conceptualizations of psychopathy are not homogenous, and definitions vary between different theoretical frameworks, sociocultural settings, and judicial systems, leading to distinctions in both academic and clinical domains [9]. In addition, the causes and determinants of psychopathic traits are still debated [1] and, although it can share certain empirical elements of antisocial and dissocial personality disorders, psychopathy is not interchangeable with these [1,10]. Similarly, psychopathy is also conceptually divergent from sociopathy [11] and may have distinct neurological and socio-behavioral influences [12].

Whilst people exhibiting moderate to high levels of psychopathic traits constitute a small percentage of the general population, evidence indicates that they may be responsible for a significant amount of misconduct, which can encompass criminal offending [13,14]. Notably, albeit an imperfect estimate, scholars have approximated that in North America, such individuals may be fifteen to twenty-five times more likely to commit crimes that lead to their incarceration [14]. This is particularly relevant for acts of violent and sexual offending [15,16]. Equally, decisions to engage in egocentric, manipulative behaviors influenced by psychopathic traits may co-occur with other high-risk activities, like substance use [17], which can further exacerbate delinquency trends.

In contrast, in some circumstances, moderate presentations of psychopathic traits can coincide with high levels of achievement [18]. Albeit challenging to define and again conceptually debated, this phenomenon, known as “successful psychopathy”, involves situations where a person is able to achieve advantageous or even superlative outcomes in their chosen field [5,13]. For example, charismatic behaviors related to psychopathic traits can include displays of confidence, an absence of anxiety, and an ability to attract positive attention and admiration [5]. Likewise, fearless dominance may be an adaptive psychopathic trait that can contribute to seemingly successful outcomes [18,19]. Additionally, individuals may feel a sense of general wellbeing, as psychopathology does not automatically preclude a sense of mental wellbeing and vice versa [20,21]. In this regard, some have argued that traits associated with “successful psychopathy” can be protective factors for antisocial behaviors, whereas other researchers have highlighted their beneficial effects solely in the short-term, which can still entail maladaptive outcomes for wider society (e.g., [22]).

Thus, dependent on varying factors, psychopathic traits can have wide-ranging implications [23,24]. For instance, there has recently been a burgeoning interest in the effects of psychopathic traits in corporate and political environments, alongside other occupational and social domains (e.g., [25,26,27]). Simultaneously, there has been an increasing emphasis on symptoms of mental disorders in sports and certain personality factors amongst athletes, which has in part been driven by the burgeoning subspeciality of sports psychiatry and advancements in sports psychology and sports medicine [28,29]. Nevertheless, to the authors’ knowledge, there has been limited awareness and sensitivity towards psychopathic traits in sporting settings across sports-psychiatric studies, forensic psychiatric and the psychological literature, and beyond. This is significant since sport represents an area of sociocultural interest involving numerous stakeholders and attracting public attention [30].

In sporting contexts, grandiosity, dominance, and aggression may manifest as hypercompetitiveness and could thereby theoretically contribute to athletic success or be injurious for other stakeholders [1,31]. For example, as noted elsewhere, athletes who cultivate and publicly share a sense of personal glory may exhibit DT traits as they perform in a way that enhances their self-esteem but could contravene ethical norms of “fair play” or sporting regulations [32]. It may also follow that an athlete’s ability to remain singularly focused on the task of training while commanding respect and creating confidence could be consistent with DT traits, as has been suggested elsewhere [33]. Welsh and Lenzenweger hypothesize that one aspect of achieving success despite exhibiting psychopathic traits is charisma, an attractive charm that is both intrinsic to the person and simultaneously appreciated by those with whom they interact [5]. Charismatic behavior can encompass displays of confidence, an absence of anxiety, and the ability to draw positive attention and admiration. As has been previously observed, it could be argued that these appear to be broadly in line with the characteristics associated with world class athletes [34]. Equally, similar paradigms could be evident in non-athlete demographics involved in competitive sports, like coaches and spectators [34]. For the latter, athletes are often considered to be aspirational role models and studies indicate that fan behaviors during sporting matches may emulate that of players [35]. Additionally, the inter-relational aspects of the athlete–coach relationship could contribute to the development and/or expression of certain characteristics, which may have multifaceted implications [36]. Hence, taken together, elevated levels of psychopathic traits could have complex outcomes in sporting environments for athletes, coaches, and spectators.

Complicating this knowledge gap further are the often-sensationalistic media portrayals of the behaviors of athletes and coaches, where psychopathic traits have been speculatively commented on by non-experts, often with pejorative language (i.e., “psycho”). For example, this has been relevant in the discourse around Nick Kyrgios, Max Scherzer, Lance Armstrong, and Ken Dorsey [37,38,39,40]. As conjecture about the psychopathology and behaviors of prominent figures (including athletes) has been described as potentially unethical and irresponsible, this could adversely shape public understanding and may even perpetuate erroneous representations about the presence and effects of psychopathic traits in sports [41,42].

Accordingly, given these considerations and the complex jurisdictional frameworks surrounding sporting competitions [43], we sought to provide a comprehensive synthesis of current evidence about this topic. Specifically, we focused on the forensic and medicolegal implications of psychopathic traits in sporting environments, alongside their potential influence on competitive drive and athletic success.

## 2. Methodology

In August 2023, we performed a narrative review of the available evidence on psychopathic traits in sport. We searched for the pertinent literature indexed in three academic databases, namely: Scopus, APA PsychNet, and PubMed.

Our search strategy was based on the following keywords: “psychopathy”, “dark triad”, “sport”, “athlete”, “coach”, “spectator”, and “fan”. Following this, we restricted our results to only include academic articles published in English and omitted book chapters, studies involving samples of children and adolescents, articles where full-text access was not available, and research that utilized sport and/or exercise as adjunct to treatment protocols for mental health disorders. We did not include a timeframe within our search methodology.

After collating applicable papers, we screened bibliographies and added further relevant articles. Following this, we removed duplicate results and those that did not contain primary empirical observations (e.g., review articles or editorials).

## 3. Results

Based on our search criteria, we identified *n* = 24 papers that discuss psychopathic traits in sporting contexts. In Supplementary Materials, a summary is presented of the bibliographic details, sample description, and instrumentation of the papers identified in the review (see Table S1 for more details [31,33,44,45,46,47,48,49,50,51,52,53,54,55,56,57,58,59,60,61,62,63,64,65]).

Based on this, we thematized these findings around specific populations, namely athletes, coaches, and spectators for readability and structure. Out of these *n* = 24 articles, *n* = 16 focused on athletes and *n* = 8 included sport adjacent non-athletes across both amateur and elite levels. This latter group consisted of *n* = 3 samples involving coaches, *n* = 3 samples of spectators present at a sporting event, and *n* = 2 samples of spectators watching a sporting event on television.

The outcomes from the studies in our review are outlined discursively below.

### 3.1. Athletes

The majority of the reviewed studies included discussions of psychopathy in athletes of varying types (i.e., collegiate, amateur, and elite) and genders, as well as those participating in both individual and team contexts.

Stanger et al. used tools like the Self-Report Psychopathy scale III (SRP-III) to understand if gender, type of athletic event, and level of competition might be correlated with DT traits in *n* = 506 medium and high-contact sport athletes [44]. In this work, males had higher psychopathy scores in game events versus record events (*p* = 0.063, d = 0.52) [44]. Males also had higher psychopathy scores versus women for game-style events (*p* = 0.086, d = 0.20) [44].

Per an investigation by Nicholls and colleagues, the relationship between the DT, athlete expertise, and mental toughness was assessed in *n* = 285 competitive athletes using instruments like the Short Dark Triad (SD3) questionnaire [45]. Psychopathy (*r* = −0.26, *p* < 0.05) and Machiavellianism (*r* = −0.30, *p* < 0.05) were negatively correlated to mental toughness, but narcissism was positively correlated (*r* = 0.82, *p* < 0.01) [45]. Results indicate that mental toughness may mediate the relationship between the DT traits and the intensity of physical activity [45].

In another study on *n* = 506 athletes from forty-two different disciplines, instruments were also used to understand if gender, type of athletic event, and level of competition might be correlated with DT traits [33]. Two and three-way interactions among DT scores and sub-scale scores for Machiavellianism, psychopathy and narcissism revealed that there were significant main effects of all three parameters [33]. In particular, males had higher psychopathy scores in game events versus record events (*p* = 0.063, *d* = 0.52) [33]. Males also had higher psychopathy scores versus women for game-style events (*p* = 0.086, *d* = 0.20) [33].

Additionally, Vaughn et al. used the Mental Toughness Questionnaire-48 with other instruments to understand the relationship between the DT, athlete expertise, and mental toughness [46]. Psychopathy (*r* = −0.26, *p* < 0.05) and Machiavellianism (*r* = −0.30, *p* < 0.05) were negatively correlated to mental toughness, but narcissism was positively correlated (r = 0.82, *p* < 0.01) [46]. Results suggested that mental toughness may mediate the relationship between the DT and the intensity of physical activity [46].

In a sample of *n* = 241 endurance athletes, the relationship between grit and DT as they relate to the development of exercise addiction was assessed (EXA) [47]. Specifically, male athletes showed more EXA, narcissism, and psychopathy [47]. There was also an inverse relationship between perseverance in effort and psychopathy (*r* = −0.43, *p* < 0.005) [47].

Furthermore, González-Hernández and colleagues assessed the relationship between DT and gender, sport type, expertise level in *n* = 1258 athletes using the SD3 [48]. For scores of narcissism, Machiavellianism, psychopathy and a composite Dark Triad score, males scored higher than females, elites scored higher than non-athletes, and individual athletes scored higher than team athletes [48].

Elsewhere, strong correlations between DT and competitiveness were found in *n* = 806 athletes of varying disciplines [49]. Differences between professional and amateur athletes were discussed in this study [49]. In particular, psychopathy predicted competitiveness (β = 0.52, *p* < 0.001), which suggested an effort to avoid failure and internal feelings of inferiority [49].

Exploring the relationship between DT and cheating and doping attitudes, a study assessed *n* = 164 athletes using instruments like the Short-Form Performance Enhancement Attitude Scale (SF-PEAS) [50]. In this research, all DT traits were positively correlated with doping attitudes [Narcissism (β  = 0.19, *p*  < 0.01), Machiavellianism (β  = 0.05, *p*  < 0.01), and psychopathy (β  = 0.44, *p*  < 0.001)] and cheating behavior [Narcissism (β  = 0.22, *p*  < 0.01), Machiavellianism (β  = 0.15, *p*  < 0.01), and psychopathy (β  = 0.12, *p*  < 0.01)] [50].

In a different work, instruments like the Multidimensional Competitive Orientation Inventory (MCOI), and basketball free throws were used to investigate the relationship between DT and sport performance. Machiavellianism (β = 0.10, *p* < 0.01), narcissism (β = 0.14, *p* < 0.01), and psychopathy (β = 0.08, *p* < 0.01) were positively related to a hypercompetitiveness. Hypercompetitiveness was positively related to free throw performance (β = 0.13, *p* < 0.01) [31].

Methods from a study by Vaughan and Madigan explored the relationship between perfectionism and dark personality traits in the risk of developing EXA in *n* = 426 runners and crossfitters [51]. For both runners and crossfitters, psychopathy had a significantly positive link with exercise addiction [crossfitters (0.03); runners (0.001) *p* < 0.05] [51]. Overall, crossfitters showed greater tendency toward psychopathy (*t*(2, 424) = 5.27; *p* < 0.001) [51].

Moreover, using instruments like the Assessment of Sadistic Personality (ASP) questionnaire, the associations between the Dark Tetrad (i.e., narcissism, Machiavellianism, psychopathy, everyday sadism) and athletic aggression were examined in *n* = 811 athletes [52]. All DT traits were positively correlated to athletic aggression, but psychopathy (β = 0.21, *p* < 0.001) and everyday sadism (β = 0.27, *p* < 0.001) had the most robust correlation [52].

Separately, instruments were used to interrogate the links between creativity constructs and DT in *n* = 364 college-age athletes [53]. Within these results, a relationship was observed between grandiose narcissism and sports, as well as a strong relationship between psychopathy and sports (r = 0.31, *p* < 0.001) [53]. Zamani Sani et al. used instruments like the Moral Content Judgement in Sport Questionnaire (MCJSQ) to study the association between DT traits and insomnia in athletes [54]. The presence of the trait psychopathy, specifically, was directly related to insomnia [r = 0.180, *p* < 0.01] [54]. Psychopathy (β = 0.194, *p* < 0.001) and ethical judgements (β = 0.156, *p* < 0.002) were found to be predictive of insomnia [54].

Other research identified within the review directly assessed athletes using anabolic steroid (AAS) use. In one such study, instruments were used to assess anabolic steroid (AAS) use and links to psychopathology in *n* = 122 gym athletes [55]. In this work, AAS users were significantly more likely to report current and former psychiatric diagnosis (*n* = 9/31%, 29.0%) than non-users (N = 4/91%, 4.4%) (*p* = 0.002), specifically with narcissistic (*n* = 5) and antisocial (*n* = 3) personality disorders [55]. Hauger and colleagues used instruments such as the antisocial personality subscale from The Millon Clinical Multiaxial Inventory-III (MCMI-III) to explore the relationship between AAS exposure, aggression, and violence in weightlifters [56]. A significantly higher percentage of those dependent on AAS (23.7%) scored over the clinical score for antisocial personality disorder, compared to those not dependent on AAS (10.7%) and those who never took AAS (1.6%), (X2 = 12.79, *p* = 0.002) [56]. Finally, another reviewed study assessing athletes who use AAS, instruments and questionnaires were implemented to understand the relationship between risk-taking behaviors and psychopathic tendencies [57]. Compared to those who did not consume AAS, individuals who used AAS had twice the odds of exhibiting psychopathic traits (OR = 2.50, 95% CI 1.52–4.15) [57]. Compared to those who did not use AAS and had never ever considered using this substance, nonusers who had considered using AAS had higher odds of exhibiting psychopathic traits (OR = 2.19, 95% CI 1.27–3.87) [57]. Further, in this work, individuals who used AAS had a 19% increase in the odds of exhibiting psychopathic traits for each additional performance-enhancing drug used (OR = 1.24, 95% CI 1.12–1.38) [57].

### 3.2. Coaches

We reviewed *n* = 3 studies that focused on coaches. In a study by Bryan et al. instruments like the Competitive Aggressiveness, and Anger Scale (CAAS) were used to assess *n* = 420 athlete–coach pairs [58]. For the athlete participants, psychopathy predicted anger and aggression [58]. DT predicted 20% of the aggression variance (F(3, 220) = 18.28, *p* = 0.001) and 14% of the variance in anger variance (F(3, 220) = 12.25, *p* = 0.002) [58]. Actor effects were found between athlete–coach pairs such that they were able to predict their own DT traits [58]. Partner effects, where athlete–coach pairs could predict each other’s DT traits were also observed [58].

In the first in a series of studies by Cook and colleagues, tools were adopted to predict coaching success and DT traits, assessing the differences in psychological characteristics between *n* = 36 world-leading and world-class swimming coaches [59]. For the Dark Triad Dirty Dozen (DTDD) instrument, significant differences were found in Machiavellianism, F(1, 34) = 5.39, *p* = 0.026, η2 = 0.137, and narcissism, F(1, 34) = 7.79, *p* = 0.009, η2 = 0.186, but not psychopathy, F(1, 34) = 2.78, *p* > 0.05, η2 = 0.076 [59].

In the second study by Cook and colleagues, similar instruments were used to investigate *n* = 38 athletes’ perceptions of psychological characteristics of world-leading vs. world-class swimming coaches [60]. Significant group differences were found in narcissism, F(1, 36) = 7.43, *p* = 0.01, η2 = 0.17, but not Machiavellianism, F(1, 36) = 3.74, *p* = 0.06, η2 = 0.09, or psychopathy, F(1, 36) = 0.06, *p* = 0.81, η2 = 0.002 [60].

### 3.3. Spectators

Several studies (*n* = 5) included discussions of psychopathy in spectators and fans who participate indirectly in sporting events, either in sporting venues or from home.

In the first of three studies by Russell and colleagues, instruments like the assault subscale of the Buss Durkee Hostility Inventory (BDHI) were used to examine motivations for participation in crowd aggression in *n* = 395 hockey fans [61]. Psychopathy and assaultiveness accounted for attraction to fights and subjects’ self-reported likelihood of involving themselves in a disturbance F(2, 63) = 14.03, *p* < 0.005 [61]. People attracted to violence expressed strong positive tendencies toward psychopathic and antisocial personality [61].

In the second study from this group on *n* = 78 hockey fans, the likelihood of participation in crowd aggression using anger and aggression was assessed using instruments such as the Grush Impulsivity Scale (GIS) and the Thrill and Adventure Seeking and Boredom Susceptibility subscales of Zuckerman’s Sensation Seeking Scale (ZSSS) [62]. In particular, psychopathy was positively correlated with a likelihood to participate in crowd aggression (β = 0.30, *p* < 0.005) [62].

In their third study, instruments were used to uncover what makes one intervene when a crowd becomes disorderly in *n* = 74 general sport spectators [63]. Sensation seeking (β = −0.03, *p* < 0.001), Impulsivity (β = −0.09, *p* < 0.001)), psychopathy (β = −0.18, *p* < 0.001), and physical aggression (β = −0.02, *p* < 0.001) were all found to be negatively correlated to peacekeeping [63].

Elsewhere, Yoder and colleagues used the Psychopathic Personality Inventory-Revised (PPI-R) and brain scans (i.e., Philips Achieva 3T) to explore the amygdala response in *n* = 43 people viewing mixed martial arts (MMA) matches selected for their violent nature [64]. Fearless Dominance and Self-Centered Impulsivity was found to be related to basolateral amygdala connectivity, suggesting that the viewers were less morally concerned and less sensitive to violence, which was related to psychopathy [64].

In an additional study, instruments like the Direct Sadism Scale and Measures of Criminal Attitudes and Associates, part B, were implemented to investigate the connection between criminal behavior, dark triad traits, and football fandom in *n* = 246 football fans [65]. Psychopathy predicted entitlement (*r* = 0.19, *p* < 0.01), antisocial intent (*r* = 0.13, *p* < 0.05), and criminal attitudes (*r* = 0.11, *p* < 0.05), but was not found to be a characteristic of the fans that explained criminal involvement [65].

## 4. Discussion

Our results show empirical findings within the reviewed literature relating to psychopathic traits in sporting frameworks and their potential forensic, medicolegal, and societal effects. These all provide a more thorough understanding of this phenomenon and raise salient discussion points within sporting contexts, especially in comparison to evidence from the general literature on psychopathic traits. Accordingly, following the demographic groupings in our review, these are categorized below based on primary actors (athletes, coaches and athlete–coach dyads, and spectators/fans), together with an overview of their implications for regulators, the media, and additional stakeholders.

### 4.1. Athletes

Per our results, psychopathic traits may engender a “win-at-all-costs” or an “anything it takes” attitude in athletes, which could have significant individual consequences and affect wider stakeholders. Firstly, in accordance with previous conceptualizations of “successful psychopathy” in general scientific studies, the literature on athletes contained in our review provides insights into how this paradoxical construct may present in sporting contexts.

DT traits have been linked to “mental toughness”, including a sense of resilience, the ability to perform under pressure, superior concentration, and unwavering self-belief [46]. While usually connected with positive characteristics like resilience and problem-solving [66], “mental toughness” in the context of DT traits may devolve into an overly rigid adherence to goals, overtraining, injury, and suppression of emotions [67]. Rigid adherence to goals in a sporting setting can be associated with exercise addiction (EXA), a psychological response which, as illustrated by Noguiera et al. in our results [47], can be impacted by DT traits. In this regard, an inclination to focus on achieving the utmost skill and ability with scant recognition of prudent training methods or potential vulnerabilities for personal injury may render athletes at any level at-risk for developing EXA; again, this association was identified by studies contained in our review (e.g., [47,51]).

Comparably, “mental toughness” can often entail a relative insensitivity to physical and psychological norms that may be required in order for an athlete to overcome barriers [68]. As indicated by the paper from Stanger et al. in our results, which found that certain athletes were less sensitive to certain unpleasant stimuli, this may lead athletes to become indifferent to sensations of their own pain or overlook the pain of others [44]. It is conceivable that such behaviors could extend outward to teammates and rivals or make athletes susceptible to illness and injury, as neglecting the prospect of physiological harm could lead to negative health-related outcomes [45]. Moreover, as discussed by the reviewed study by Greitemeyer, rivalry in the context of DT traits may present as antagonistic versus admiring [52], which could trigger fraught interactions with other athletes and serve as a justification for foul play [69].

Analogously, the desire to do “anything it takes” to succeed can be considered a positive feature of participation in sport, though, notably, this trait is a component of Levenson’s Self-Report Psychopathy Scale [70]. Likewise, in line with prior theories on “successful psychopathy” in the general literature, the reviewed studies demonstrate the presentation of DT traits associated with aggression and competitiveness, which concurrently may contribute to athletic success [35]. The appropriateness of aggression in sport is a contentious issue, since it can allow for both energetic achievements during play but may concurrently stimulate violence [71]. That said, it should be noted that competition is conventionally accepted as a necessary feature of sport and sportsmanship [72]. It therefore could follow, as work in our review suggests, that certain traits consistent with moderate psychopathy may be advantageous in certain sporting contexts, as they are connected to, and likely aid with, the development of skills and demonstration of athletic ability [31].

Nevertheless, as discussed in the work by Ueno et al., the presence of DT traits can lead athletes to act aggressively and disregard the wellbeing of others within sporting environments [33]. Conceivably, fluctuating expressions of DT traits could contribute to success in a variety of sports, whether they require physical aggression or not, as violence is not always a conditional feature of models of psychopathy [73]. Using a Dark Tetrad model, the work by Greitemeyer in our review considers the implications of harm to others in sport with the inclusion of “everyday sadism”, that being distinct from “Coercive Sexual Sadism Disorder” as recognized in the *International Classification of Disorders 11th Revision* [52]. In the authors’ opinion, the relationship between psychopathic traits and physical aggression indicated by studies in our results may be concerning since there is evidence of elite athletes simultaneously behaving violently “off the field”, resulting in criminal charges and judicial issues [43,74].

Further, it is possible that an “anything it takes” mentality in the context of psychopathic traits could engender other adverse medicolegal consequences, including doping behaviors, fraud, sexual assault, or violent offending [42,45,47]. Specifically, according to the study by Nelson et al. in our review [57], anabolic steroid (AAS) use in those exhibiting psychopathic traits has correlations with risky sexual behavior, impulsivity, and anger problems. These findings in a sporting setting align with other scholarly literature in different contexts that links psychopathic traits with sexual aggression [75].

Akin to this, evidence from our review suggests that the presence of traits linked to psychopathy could contribute to the decision to use performance enhancing drugs (PEDs), such as AAS, which can have serious (even fatal) health consequences, and may exacerbate aggression and violence [17,55,76]. As evidenced by the reviewed study by Nicholls et al. where it was found that athletes scoring higher on DT traits are more likely to use PEDs, the rejection of psychological norms to achieve performance goals could lead to the breaking of societal conventions, the circumvention of legal frameworks, and athletes discounting the threat of being caught for substance use transgressions [45].

In the authors’ view, when maladaptive behaviors are revealed, these effects may not only be problematic for the individual but also could entail harms for those who share toxic personal and professional relationships with the athlete. A recent primer by DeBrito et al. discussed people exhibiting severe psychopathic traits using manipulation in a targeted way [11]. As both the incidence of doping and the variety of PEDs available appear to be increasing, these aspects may require additional attention and academic scrutiny [77,78].

Moreover, as *n* = 3 papers in our review indicate, exposure to drugs like AAS may further contribute to the exhibition of behaviors consistent with high levels of psychopathic traits. Although research remains inconclusive, particularly in relation to causal temporality, certain correlations between AAS and the detrimental brain effects and presentation of psychopathic traits have been previously proposed in other studies [56,79].

### 4.2. Athlete–Coach Dynamics

From the evidence shown in our review, the relationship between coaches and athletes is an additional area of concern when psychopathic traits manifest, particularly in situations where the athlete or team has been less successful.

Significantly, the reviewed papers demonstrate that coaches have been shown to exhibit more DT traits in world-class sporting contexts [59,60]. However, as per the study by Cook, Fletcher, and Peyrebrune, the perception of psychopathic traits in coaches by their athletes does not seem to correlate with relative coaching success in upper echelons of sport [59].

It is important to note that findings in *n* = 2 of the studies, athletes’ perceptions of coach behavior were measured without assessing DT traits within those athletes or how they would affect those perceptions [59,60]. This may be significant as research indicates that DT traits are shared among athlete–coach pairs and that aggression and violence are closely linked to these traits [58]. In particular, per the reviewed study by Bryan et al., in which interdependence models were used to analyze DT traits, unprovoked violence could be a concern in coach–athlete pairs where psychopathy is a factor [58].

Studies from sports psychiatry have shown that physical and psychological abuse towards athletes is becoming a major area of focus, which is leading national agencies and regulators to consider appropriate actions and safeguarding policies [80]. Additionally, the use of PEDs is more likely within these athlete–coach pairs [58]. Again, we believe that per our findings, the presentation of psychopathic traits requires more detailed investigation within these contexts, especially since psychopathic traits may influence such behaviors.

Moreover, evidence from the general literature shows that people exhibiting DT traits may look for certain characteristics in romantic partners that would allow them to optimize these traits and/or continue to exhibit them unabated [81]. In addition, outside of the sports medicine literature, psychopathic traits have been linked to coercive behaviors in romantic partners [82]. While based on the studies in our review, we cannot speculate on how coaches with DT traits choose to work with certain athletes, we do acknowledge that the exhibition of maladaptive behaviors on teams or in solo sports could create difficulties such as increased anxiety, pressure, and emotional tensions within the dyad [83]. This could again form the foundation for future inquiries in sports medicine, sports psychiatry, forensic studies, and beyond.

### 4.3. Spectators

Concerningly, as per our results, fans and spectators may also exhibit similar behavioral profiles as the athletes that they are observing, which aligns with findings from elsewhere (e.g., [84]). Aligning oneself with a specific team or athlete can carry strong cultural and social value, creating a sense of identity and belonging [35,85]. As demonstrated in the reviewed literature, if the team or athlete performs poorly or is otherwise subject to negative outcomes during play, spectators could conceivably experience co-occurring feelings ranging from shame, anger, and regret, to disgust, alienation, and failure, which may contribute to aggression and violence [35,61,62,63]. Together with this, other academic findings also support the notion that fans identify deeply with their preferred team (e.g., football fans) [86]. As there has been an increase in criminal activity and sport-related hooliganism [87], these findings may require increased interprofessional collaborations and further attention [88].

As outlined in reviewed studies on both hockey and MMA fans, in certain situations, a spectator exhibiting psychopathic traits may not be emotionally invested in the outcome of the sporting match but are more engaged with the spectacle of violence itself [61,64]. Witnessing violent acts may further encourage violent behavior and it has been speculated that fans may attend particular sports expressly for the purpose of watching fights, as evidenced by the reviewed study by Russell on hockey fans [61]. As such, results from our review shows that DT traits are intrinsically present in certain fans and observers of sport a priori [61,63,64], which has also been theorized elsewhere in the literature on fan violence in soccer [89].

Interestingly, research has found that types of fans seem to exhibit violence in absence of the manipulation and deception normally associated with psychopathic traits [65]. It has been suggested that this may be due to the complexities of how psychopathy may render one indifferent to emotions, but likely to engage in unprovoked violence [65]. Again, this phenomenon could have wider significance beyond sports psychiatry and forensic psychiatry and psychology, requiring multidisciplinary collaborations.

Separately, as both Monaghan [90] and Pappa and Kennedy [91] posit, PED use can be predicated upon the normalization of these substances in their broader respective communities. As shown by work on drug policy and sports medicine, PED use has become more frequent in amateur athletes across different sporting disciplines owing to various reasons like enhanced self-image and body composition improvements [92,93]. Nevertheless, we do not discount the potential influence that athlete doping could have on usage trends in the recreational sporting public with similar forensic interactions and adverse health outcomes [90,91].

### 4.4. Media Representations and Regulatory Awareness

Given the results of our study and the general literature on psychopathic traits, we believe there are additional responsibilities for various stakeholders including the media and regulators. Firstly, it is our view that athletes and coaches exhibiting behaviors consistent with psychopathy, whether or not traits are present, may be vulnerable to media sensationalization, as has been implied by reporting on both team and individual disciplines [37,38,94].

For us, the charismatic personality that research suggests is typical of those high achieving athletes exhibiting psychopathic traits may conceivably draw media and public attention [34]. In turn, this sensationalist reporting could nullify the potentially grave nature of psychopathic traits in certain contexts and might invoke correlations between adversarial actions and athletic success without caveat [95]. Additionally, the normalization of hostile behaviors in sports through media reporting could further exacerbate this issue, preventing stakeholders from recognizing the need for appropriate actions [96,97]. In this regard, academic inquiries indicate that outlandish media coverage often reinforces stigma in the general public, which could potentially cause alienation and low self-esteem [98] and may be relevant in sporting contexts.

As behaviors consistent with psychopathy in sporting settings are likely to play out in public and be discussed by others in open forums [35], we believe that measures should be taken to adequately contextualize these concerns in popular discourse. Thus, we contend that media outlets should refrain from referring to athletes and coaches with pejorative language that would disrespect and dehumanize the individual. This is particularly important given recent evidence around the influence of the media in shaping popular opinions on professional athletes, often leading to negative perceptions and/or contributing to additional deviant behaviors [99]. Accordingly, we suggest that there should be further ethical consideration towards these sensationalistic depictions for the purpose of drawing additional interest to a sport or sporting event, particularly when financial stakeholders stand to benefit; in a general sense, Wahl has noted that hyperbolic media reporting is often driven by profit [100].

From a regulatory perspective, we recognize the efforts of major governing bodies to offer psychological support to professional athletes and stakeholders, creating awareness about general psychopathological issues [101,102,103]. As psychopathy is a dimensional and complex construct [11], we also acknowledge the challenges amongst the general population or non-experts associated with perceiving psychopathic traits as the reason for maladaptive behaviors.

However, according to the general literature and the results from our review, individuals who exhibit DT traits often behave in ways that are contrary to moral, ethical, and legal norms [1,58,103]. In such cases, we encourage regulators and teams to seek expert opinions, especially when there are indications that larger networks of stakeholders have been manipulated into fraudulent behaviors.

More broadly, we strongly urge regulatory bodies to mitigate risks by establishing zero tolerance policies around sexual misconduct, violence, coercion, or any type of harassment or manipulation. Strong policies around antidoping, fraud or manipulation of match outcomes, and fair play in general would also be beneficial.

## 5. Limitations and Future Research Directions

We deemed our methodology to be an appropriate method to synthesize current evident in this area, though it is not without its limitations. Firstly, our search uses a narrative methodology, as this allowed for critical decision-making around the inclusion or exclusion of evidence, which was particularly important since conceptualizations of psychopathic traits can be heterogeneous [104,105]. Further, in an effort to include critical though older data we did not limit the timeframe for our search, which may have caused us to inadvertently include outdated evidence [106]. Nevertheless, this was intended to capture a broad evidence base given the paucity of studies in this area; similar strategies have been adopted in previous reviews examining certain areas of psychopathology in sport where research can be limited [107]. By confining our search to Scopus, APA PsychNet, and PubMed, we could have omitted studies that would not be captured within these databases or the grey literature. Equally, our results only show papers where we had full-text access to the article meaning that certain evidence will not have been captured.

With regard to evidential quality, 100% of the studies in our review (*n* = 24) used self-report psychometric questionnaires such as the BFI, LSRP, and PPI-R to assess psychopathic traits in participants. In addition, five studies (*n* = 5) used unique self-report questions to gain a retrospective account of subjects’ habits. Though self-report is a valuable method of capturing data, it does rely on individual perspective and is limited by personal biases in retrospective contexts [108]. Sample sizes were generally robust among athletes and *n* = 7 studies involved a mix of amateurs and elite/professionals across various sports. Of these, highlighting the coach–athlete dyad, *n* = 2 studies specified that they included solely elite athletes [59,60]. The sample sizes in these latter studies were small, likely due to the difficulty in recruiting such pairs at the world-class level [109].

There is an inherent gender bias in the papers that we investigated, as there may be within the study of psychopathy more broadly [110]. Notably, across four studies, inquiries into crowd behavior focused only on men at a hockey game, men as general sport spectators, and male football fans [61,62,63,65]. Equally, all of the reviewed research on AAS use involved men, though the psychiatric and behavioral concerns of females who use AAS have been illustrated elsewhere [111,112]. That said, there appears to be a general absence of forensic inquiry into AAS use in women, despite observed links to criminal behavior [113]. In one example utilizing imagery of MMA fighters to elicit emotional responses from the viewer, a male-only sample was recruited based on the reasoning that only men watch violent sports [64]. While this study elicited interesting results, we believe that researchers should avoid stereotyping in order to highlight these developing intersections, especially given the growing presence of female athletes [114] and female spectators [115] internationally.

Generally, future research on psychopathic traits in sport is necessary to increase knowledge across sports medicine, sports psychiatry, sports psychology, forensic fields, and other domains. That said, we do acknowledge the difficulty of recruiting subjects for studies on psychopathy and also in sport, which may contribute to the lack of empirical evidence in this area [116,117,118]. Accordingly, it is likely that existing evidence does not fully represent and may likely underestimate the prevalence of psychopathic traits in all sporting settings; in this regard, self-report ratings could contribute to this problem. This follows the existing literature that highlights the difficulty in accurate reporting of psychopathic traits [119]. However, the reviewed studies show that the expression of behaviors associated with psychopathy were evident in athletes of various disciplines, alongside coaches and spectators.

The extant literature does not address whether certain sporting contexts are more likely to have a symbiotic relationship with specific psychopathic traits, and/or the expression of psychopathic traits overall. Separately, emerging research on the relationship between traumatic brain injury (TBI) and psychopathic traits supports the need for additional study and discussions around sports with a high risk for TBI and abnormal athlete behaviors [120]. Finally, implicit in the concept of “successful psychopathy” is the potential that those who exhibit psychopathic traits are successful in their sporting endeavors. Nevertheless, our review shows a lack of evidence into whether those athletes exhibiting psychopathic traits win more matches or achieve more success due to advantages that are not associated with sporting skills.

## 6. Conclusions

We performed a narrative review examining psychopathic traits in sport. Our results indicate that psychopathic traits and behaviors consistent with these traits may be present in athletes, coaches, and spectators. Our findings not only highlight forensic implications resulting from this, but that interrelated medicolegal, societal, cultural, and ethical concerns must be addressed in all sporting contexts. Conversely, certain papers included in our review show that psychopathy can entail beneficial aspects for sporting achievement, following recent inquiry into “successful psychopathy” in other contexts. As sport has deep sociocultural significance, psychopathic traits may likely have a great influence on wider public perceptions; again, this was demonstrated by papers in our review. Accordingly, we believe that more studies and attention must be directed toward this topic. The often sensationalist media portrayal of these complex traits should be discouraged, as it may increase stigma and decrease opportunities for the management of mental health disorders. Moreover, robust policies and interventions should be considered to protect athletes, coaches, fans, and the entire sporting community from the negative effects of psychopathic traits.

## Supplementary Materials

The following supporting information can be downloaded at: https://www.mdpi.com/article/10.3390/sports11110228/s1, Table S1: Studies Discussing Psychopathy in Sporting Contexts.

## References

[1] Hare R.D., Neumann C.S. (2009). Psychopathy: Assessment and Forensic Implications. Can. J. Psychiatry.

[2] Rauthmann J. (2012). The Dark Triad and Interpersonal Perception: Similarities and Differences in the Social Consequences of Narcissism, Machiavellianism, and Psychopathy. Soc. Psychol. Personal. Sci..

[3] Hemphill J.F., Hart S.D. (2003). Forensic and Clinical Issues in the Assessment of Psychopathy. Handbook of Psychology: Forensic Psychology.

[4] Anderson N.E., Kiehl K.A. (2014). Psychopathy: Developmental Perspectives and Their Implications for Treatment. Restor. Neurol. Neurosci..

[5] Welsh E.-C.O., Lenzenweger M.F. (2021). Psychopathy, Charisma, and Success: A Moderation Modeling Approach to Successful Psychopathy. J. Res. Personal..

[6] Furnham A., Richards S.C., Paulhus D.L. (2013). The Dark Triad of Personality: A 10 Year Review. Soc. Personal. Psychol. Compass.

[7] Paulhus D.L., Williams K.M. (2002). The Dark Triad of Personality: Narcissism, Machiavellianism, and Psychopathy. J. Res. Personal..

[8] Muris P., Merckelbach H., Otgaar H., Meijer E. (2017). The Malevolent Side of Human Nature: A Meta-Analysis and Critical Review of the Literature on the Dark Triad (Narcissism, Machiavellianism, and Psychopathy). Perspect. Psychol. Sci..

[9] Brooks N., Fritzon K., Brooks N., Croom S. (2020). Conceptualising Psychopathy: Empirical, Clinical and Case Interpretations. Corporate Psychopathy: Investigating Destructive Personalities in the Workplace.

[10] Abdalla-Filho E., Völlm B. (2020). Does Every Psychopath Have an Antisocial Personality Disorder?. Braz. J. Psychiatry.

[11] De Brito S.A., Forth A.E., Baskin-Sommers A.R., Brazil I.A., Kimonis E.R., Pardini D., Frick P.J., Blair R.J.R., Viding E. (2021). Psychopathy. Nat. Rev. Dis. Primers.

[12] Pemment J. (2013). Psychopathy versus Sociopathy: Why the Distinction Has Become Crucial. Aggress. Violent Behav..

[13] Babiak P., Hare R.D. (2006). Snakes in Suits: When Psychopaths Go to Work.

[14] Kiehl K.A., Hoffman M.B. (2011). The Criminal Psychopath: History, Neuroscience, Treatment, and Economics. Jurimetrics.

[15] Hart S.D., Kropp P.R., Hare R.D. (1988). Performance of Male Psychopaths Following Conditional Release from Prison. J. Consult. Clin. Psychol..

[16] Hare R.D., Raine A., Sanmartín J. (2001). Psychopaths and Their Nature. Violence and Psychopathy.

[17] Piacentino D., D Kotzalidis G., del Casale A., Rosaria Aromatario M., Pomara C., Girardi P., Sani G. (2015). Anabolic-Androgenic Steroid Use and Psychopathology in Athletes. A Systematic Review. Curr. Neuropharmacol..

[18] Wallace L., Fido D., Sumich A.L., Heym N. (2022). A Systematic Review on the Current Conceptualisations of Successful Psychopathy. Forensic Sci. Int. Mind Law.

[19] Lilienfeld S.O., Watts A.L., Smith S.F. (2015). Successful Psychopathy: A Scientific Status Report. Curr. Dir. Psychol. Sci..

[20] Gilmour H. (2014). Positive Mental Health and Mental Illness. Health Rep..

[21] Lovelace L., Gannon L. (1999). Psychopathy and Depression: Mutually Exclusive Constructs?. J. Behav. Ther. Exp. Psychiatry.

[22] Benning S.D., Venables N.C., Hall J.R. (2018). Successful Psychopathy. Handbook of Psychopathy.

[23] Hicks B.M., Carlson M.D., Blonigen D.M., Patrick C.J., Iacono W.G., MGue M. (2012). Psychopathic Personality Traits and Environmental Contexts: Differential Correlates, Gender Differences, and Genetic Mediation. Personal. Disord..

[24] Pullman L.E., Refaie N., Lalumière M.L., Krupp D. (2021). Is Psychopathy a Mental Disorder or an Adaptation? Evidence from a Meta-Analysis of the Association Between Psychopathy and Handedness. Evol. Psychol..

[25] McCullough J. The Psychopathic CEO. Forbes.com. https://www.forbes.com/sites/jackmccullough/2019/12/09/the-psychopathic-ceo/.

[26] Palmen D., Derksen J., Kolthoff E. (2018). House of Cards: Psychopathy in Politics. Public Integr..

[27] Irtelli F., Vincenti E., Irtelli F., Vincenti E. (2017). Successful Psychopaths: A Contemporary Phenomenon. Psychopathy—New Updates on an Old Phenomenon.

[28] Piepiora P., Piepiora Z. (2021). Personality Determinants of Success in Men’s Sports in the Light of the Big Five. Int. J. Environ. Res. Public Health.

[29] Glick I.D., Reardon C.L., Stull T. (2022). Sports Psychiatry: An Update and the Emerging Role of the Sports Psychiatrist on the Sports Medicine Team. Clin. J. Sport Med..

[30] Puertas-Molero P., Marfil-Carmona R., Zurita-Ortega F., González-Valero G. (2019). Impact of Sports Mass Media on the Behavior and Health of Society. A Systematic Review. Int. J. Environ. Res. Public Health.

[31] Vaughan R.S., Madigan D.J. (2021). The Winner Takes It All: The Mediating Role of Competitive Orientations in the Dark Triad and Sport Task Performance Relationship. Eur. J. Sport Sci..

[32] Roberts R., Cooke A., Woodman T., Hupfeld H., Barwood C., Manley H. (2019). When the Going Gets Tough, Who Gets Going? An Examination of the Relationship between Narcissism, Effort, and Performance. Sport Exerc. Perform. Psychol..

[33] Ueno Y., Shimotsukasa T., Suyama S., Oshio A. (2017). Correlations between Competitive Sports’ Characteristics and the Dark Triad. J. Phys. Educ. Sport.

[34] Delaney T. (2020). Charisma in Sports. Routledge International Handbook of Charisma.

[35] Peco J., Gerin J. (2022). Sports and Violence. Sport. Logos.

[36] Stirling A.E., Kerr G.A. (2009). Abused athletes’ perceptions of the coach-athlete relationship. Sport Soc..

[37] Williams M. All the Times Nick Kyrgios Earned His “Tennis Badboy” Title. Sports Illustrated. https://www.si.com/tennis/2022/08/26/nick-kyrgios-controversial-moments-fines-timeline.

[38] Matz E. The True Madness of Max Scherzer—ESPN. ESPN.com. https://www.espn.com/mlb/story/_/id/23793792/the-true-madness-max-scherzer.

[39] Nanos J. Is Lance Armstrong A Psychopath? Boston Magazine. https://www.bostonmagazine.com/news/2013/01/22/lance-armstrong-psychopath/.

[40] Terranova J. (2022). Ken Dorsey’s Bills Meltdown Puts a New Spin on His ‘Psychopath’ Comment [Internet]. https://nypost.com/2022/09/26/ken-dorseys-bills-meltdown-puts-a-new-spin-on-his-psychopath-comment/.

[41] Steen R. (2016). Sensationalists United? Football Hooliganism and the English Press. Sport Soc..

[42] Levine M.A. (2017). Journalism Ethics and the Goldwater Rule in a “Post-Truth” Media World. J. Am. Acad. Psychiatry Law.

[43] Smith A.J., Buadze A., Claussen M.C., Seifritz E., Liebrenz-Rosenstock M. (2022). On the Same Team: A Call for Increased Medicolegal Knowledge Exchanges between Forensic Psychiatry and Sports Psychiatry. Front. Psychiatry.

[44] Stanger N., Kavussanu M., Willoughby A., Ring C. (2012). Psychophysiological Responses to Sport-Specific Affective Pictures: A Study of Morality and Emotion in Athletes. Psychol. Sport Exerc..

[45] Nicholls A.R., Madigan D.J., Backhouse S.H., Levy A.R. (2017). Personality Traits and Performance Enhancing Drugs: The Dark Triad and Doping Attitudes among Competitive Athletes. Personal. Individ. Differ..

[46] Vaughan R., Carter G.L., Cockroft D., Maggiorini L. (2018). Harder, Better, Faster, Stronger? Mental Toughness, the Dark Triad and Physical Activity. Personal. Individ. Differ..

[47] Nogueira A., Tovar-Gálvez M., González-Hernández J. (2019). Do It, Don’t Feel It, and Be Invincible: A Prolog of Exercise Addiction in Endurance Sports. Front. Psychol..

[48] Vaughan R., Madigan D., Carter G., Nicholls A. (2019). The Dark Triad in Male and Female Athletes and Non-Athletes: Group Differences and Psychometric Properties of the Short Dark Triad (SD3). Psychol. Sport Exerc..

[49] González-Hernández J., Cuevas-Campos R., Tovar-Gálvez M.I., Melguizo-Rodríguez L. (2020). Why Negative or Positive, If It Makes Me Win? Dark Personality in Spanish Competitive Athletes. Int. J. Environ. Res. Public Health.

[50] Nicholls A.R., Madigan D.J., Duncan L., Hallward L., Lazuras L., Bingham K., Fairs L.R.W. (2020). Cheater, Cheater, Pumpkin Eater: The Dark Triad, Attitudes towards Doping, and Cheating Behaviour among Athletes. Eur. J. Sport Sci..

[51] Gonzalez Hernandez J., Baños R., Morquecho-Sánchez R., Pineda-Espejel H.A., Chamorro J. (2021). Perfectionism Patterns, Dark Personality, and Exercise Addiction Trend in High-Intensity Sports. Int. J. Ment. Health Addict..

[52] Greitemeyer T. (2022). The Dark Side of Sports: Personality, Values, and Athletic Aggression. Acta Psychol..

[53] Sordia N., Jauk E., Martskvishvili K. (2022). Beyond the Big Personality Dimensions: Consistency and Specificity of Associations between the Dark Triad Traits and Creativity. Psychol. Aesthet. Creat. Arts.

[54] Zamani Sani S.H., Greco G., Fathirezaie Z., Badicu G., Aghdasi M.T., Abbaspour K., Fischetti F. (2023). Which Dark Personality Traits Could Predict Insomnia? The Mediated Effects of Perceived Stress and Ethical Judgments. Behav. Sci..

[55] Piacentino D., Sani G., Kotzalidis G.D., Cappelletti S., Longo L., Rizzato S., Fabi F., Frati P., Fineschi V., Leggio L. (2022). Anabolic androgenic steroids used as performance and image enhancing drugs in professional and amateur athletes: Toxicological and psychopathological findings. Hum. Psychopharmacol..

[56] Hauger L.E., Havnes I.A., Jørstad M.L., Bjørnebekk A. (2021). Anabolic Androgenic Steroids, Antisocial Personality Traits, Aggression and Violence. Drug Alcohol Depend..

[57] Nelson B.S., Hildebrandt T., Wallisch P. (2022). Anabolic–Androgenic Steroid Use Is Associated with Psychopathy, Risk-Taking, Anger, and Physical Problems. Sci. Rep..

[58] Bryan W., Donachie T.C., Vaughan R.S., Madigan D.J. (2023). Don’t Look Back in Anger: A Cross-Sectional and Dyadic Examination of the Dark Triad, Anger, and Aggression in Athletes. Psychol. Sport Exerc..

[59] Cook G.M., Fletcher D., Peyrebrune M. (2021). Olympic Coaching Excellence: A Quantitative Study of Psychological Aspects of Olympic Swimming Coaches. Psychol. Sport Exerc..

[60] Cook G.M., Fletcher D., Peyrebrune M. (2022). Olympic Coaching Excellence: A Quantitative Study of Olympic Swimmers’ Perceptions of Their Coaches. J. Sports Sci..

[61] Russell G.W. (1995). Personalities in the Crowd: Those Who Would Escalate a Sports Riot. Aggr. Behav..

[62] Russell G.W., Arms R.L. (1998). Toward a Social Psychological Profile of Would-Be Rioters. Aggress. Behav..

[63] Russell G.W., Arms R.L., Mustonen A. (1999). When Cooler Heads Prevail: Peacemakers in a Sports Riot. Scand. J. Psychol..

[64] Yoder K., Porges E., Decety J. (2014). Amygdala Subnuclei Connectivity in Response to Violence Reveals Unique Influences of Individual Differences in Psychopathic Traits in a Nonforensic Sample. Hum. Brain Mapp..

[65] Međedović J., Kovačević U. (2021). Sadism as a Key Dark Trait in the Link Between Football Fandom and Criminal Attitudes. J. Individ. Differ..

[66] Crust L., Clough P.J. (2011). Developing Mental Toughness: From Research to Practice. J. Sport Psychol. Action..

[67] Tibbert S., Andersen M., Morris T. The Dark Side of Mental Toughness: Subcultural Imperatives That Harm. Proceedings of the 14th FEPSAC Congres.

[68] Liew G.C., Kuan G., Chin N.S., Hashim H.A. (2019). Mental Toughness in Sport. Ger. J. Exerc. Sport. Res..

[69] Kosiewicz J. (2014). Foul Play in Sport as a Phenomenon Inconsistent with the Rules, yet Acceptable and Desirable: Ethical Conditions. Phys. Cult. Sport.

[70] Levenson M.R., Kiehl K.A., Fitzpatrick C.M. (1995). Assessing Psychopathic Attributes in a Noninstitutionalized Population. J. Personal. Soc. Psychol..

[71] Griffin M. (1996). Aggression in Sport: Inevitable, Avoidable, or a Matter of Semantics?. Peace Confl. J. Peace Psychol..

[72] Lad Sessions W. (2004). Sportsmanship as Honor. J. Philos. Sport.

[73] Walsh Z., Swogger M.T., Walsh T., Kosson D.S. (2007). Psychopathy and Violence: Increasing Specificity. Neth. J. Psychol..

[74] Withers B.P. (2015). Without Consequence: When Professional Athletes Are Violent off the Field. Harv. J. Sports Entertain. Law.

[75] Mouilso E.R., Calhoun K.S. (2012). Narcissism, Psychopathy and Five-Factor Model in Sexual Assault Perpetration. Personal. Ment. Health.

[76] Pope H.G., Katz D.L. (1990). Homicide and Near-Homicide by Anabolic Steroid Users. J. Clin. Psychiatry.

[77] Latest UKAD Testing Numbers Increase Again. UK Anti-Doping. https://www.ukad.org.uk/news/latest-ukad-testing-numbers-increase-again.

[78] Vlad R.A., Hancu G., Popescu G.C., Lungu I.A. (2018). Doping in Sports, a Never-Ending Story?. Adv. Pharm. Bull..

[79] Medras M., Brona A., Józków P. (2018). The Central Effects of Androgenic-Anabolic Steroid Use. J. Addict. Med..

[80] Schmidt R.E., Schneeberger A.R., Claussen M.C. (2022). Interpersonal violence against athletes: What we know, what we need to know, and what we should do. Sports Psychiatry J. Sports Exerc. Psychiatry.

[81] Lyons M. (2019). The Dark Triad of Personality: Narcissism, Machiavellianism, and Psychopathy in Everyday Life.

[82] Jones D.N., Olderbak S.G. (2014). The Associations Among Dark Personalities and Sexual Tactics Across Different Scenarios. J. Interpers. Violence.

[83] Diller S.J., Frey D., Jonas E. (2021). Coach Me If You Can! Dark Triad Clients, Their Effect on Coaches, and How Coaches Deal with Them. Coach. Int. J. Theory Res. Pract..

[84] Havard C.T. (2014). Glory Out of Reflected Failure: The Examination of How Rivalry Affects Sport Fans. Sport Manag. Rev..

[85] Aggerholm K., Breivik G. (2021). Being, Having and Belonging: Values and Ways of Engaging in Sport. Sport Soc..

[86] Newson M. (2019). Football, Fan Violence, and Identity Fusion. Int. Rev. Sociol. Sport.

[87] Football-Related Arrests and Banning Orders, England and Wales: 2021 to 2022 Season. GOV.UK. https://www.gov.uk/government/statistics/football-related-arrests-and-banning-orders-england-and-wales-2021-to-2022-season/football-related-arrests-and-banning-orders-england-and-wales-2021-to-2022-season.

[88] Pearson G., Stott C. (2022). A New Agenda for Football Crowd Management: Reforming Legal and Policing Responses to Risk.

[89] Kerr J. (1994). Understanding Soccer Hooliganism.

[90] Monaghan L.F. (2009). Commentary on Kanayama et al. (2009): The Normalization of Steroid Use. Addiction.

[91] Pappa E., Kennedy E. (2013). ‘It Was My Thought … He Made It a Reality’: Normalization and Responsibility in Athletes’ Accounts of Performance-Enhancing Drug Use. Int. Rev. Sociol. Sport.

[92] Henning A.D., Dimeo P. (2018). The New Front in the War on Doping: Amateur Athletes. Int. J. Drug Policy.

[93] Christiansen A.V., Frenger M., Chirico A., Pitsch W. (2023). Recreational Athletes’ Use of Performance-Enhancing Substances: Results from the First European Randomized Response Technique Survey. Sports Med. Open.

[94] Fainaru-Wada M. ESPN.com. 2015. Inside Hope Solo’s Domestic Violence Case. https://www.espn.com/espn/otl/story/_/id/12976615/detailed-look-hope-solo-domestic-violence-case-includes-reports-being-belligerent-jail.

[95] Valença A.M. (2018). Antisocial Personality Disorder, Psychopathy and Media. J. Bras. Psiquiatr..

[96] Ramaeker J., Petrie T.A. (2019). “Man up!”: Exploring Intersections of Sport Participation, Masculinity, Psychological Distress, and Help-Seeking Attitudes and Intentions. Psychol. Men Masculinities.

[97] Souter G., Lewis R., Serrant L. (2018). Men, Mental Health and Elite Sport: A Narrative Review. Sports Med—Open.

[98] Babić D., Babić R., Vasilj I., Avdibegović E. (2017). Stigmatization of Mentally Ill Patients through Media. Psychiatr. Danub..

[99] Boykoff J., Carrington B. (2020). Sporting Dissent: Colin Kaepernick, NFL Activism, and Media Framing Contests. Int. Rev. Sociol. Sport.

[100] Wahl O.F. (1995). Media Madness: Public Images of Mental Illness.

[101] USTA Launches Mental Health Initiative at 2021 US Open—Official Site of the 2023 US Open Tennis Championships—A USTA Event. https://www.usopen.org/en_US/news/articles/2021-08-24/usta_launches_mental_health_initiative_at_2021_us_open.html.

[102] (2017). Respect and Responsibility Review. https://www.nzrugby.co.nz/assets/NZR-RRR-Summary-Document.pdf.

[103] Association T.F. The Website for the English Football Association, the Emirates FA Cup and the England Football Team. http://www.thefa.com/football-rules-governance/policies/equality/mental-health.

[104] Collins J.A., Fauser B.C.J.M. (2005). Balancing the Strengths of Systematic and Narrative Reviews. Hum. Reprod. Update.

[105] Hart S.D., Cook A.N. (2012). Current Issues in the Assessment and Diagnosis of Psychopathy (Psychopathic Personality Disorder). Neuropsychiatry.

[106] Pautasso M. (2013). Ten Simple Rules for Writing a Literature Review. PLoS Comput. Biol..

[107] Colangelo J., Smith A., Buadze A., Keay N., Liebrenz M. (2023). Mental Health Disorders in Ultra Endurance Athletes per ICD-11 Classifications: A Review of an Overlooked Community in Sports Psychiatry. Sports.

[108] Pekruna R. (2020). Commentary: Self-Report Is Indispensable to Assess Students’ Learning. Frontline Learn. Res..

[109] Skorski S., Hecksteden A. (2021). Coping With the “Small Sample–Small Relevant Effects” Dilemma in Elite Sport Research. Int. J. Sports Physiol. Perform..

[110] Conley C. (2020). Examining the Effects of Rater Characteristics and Gender Biases on Ratings of Psychopathic Traits for Women. Master’s Thesis.

[111] Gruber A.J., Pope H.G. (2000). Psychiatric and Medical Effects of Anabolic-Androgenic Steroid Use in Women. Psychother. Psychosom..

[112] Cashdan E. (2003). Hormones and Competitive Aggression in Women. Aggress. Behav..

[113] Lundholm L., Käll K., Wallin S., Thiblin I. (2010). Use of Anabolic Androgenic Steroids in Substance Abusers Arrested for Crime. Drug Alcohol Depend..

[114] Stavropoulos V. Women Participation in Sports Events. Statathlon: Intelligence as a Service. https://statathlon.com/the-evolution-of-women-participation-sports-events/.

[115] As Women’s Sports Grows, So Does the Female Fan Base. Altman Solon. https://www.altmansolon.com/insights/womens-sports-and-female-fan-base/.

[116] Gulliver A., Griffiths K.M., Christensen H. (2012). Barriers and Facilitators to Mental Health Help-Seeking for Young Elite Athletes: A Qualitative Study. BMC Psychiatry.

[117] Mullins-Sweatt S.N., Glover N.G., Derefinko K.J., Miller J.D., Widiger T.A. (2010). The Search for the Successful Psychopath. J. Res. Personal..

[118] Krusemark E.A. (2011). Neurophysiological Correlates of Narcissism and Psychopathy. The Handbook of Narcissism and Narcissistic Personality Disorder.

[119] Verschuere B., Uzieblo K., De Schryver M., Douma H., Onraedt T., Crombez G. (2014). The Inverse Relation between Psychopathy and Faking Good: Not Response Bias, but True Variance in Psychopathic Personality. J. Forensic Psychiatry Psychol..

[120] Koenig S., Wu Z., Gao Y., Li X. (2020). Abnormal Cortical Activation in Visual Attention Processing in Sub-Clinical Psychopathic Traits and Traumatic Brain Injury: Evidence from an fNIRS Study. J. Psychopathol. Behav. Assess..

