# Supplementary material for: Beyond a Game: A Narrative Review of Psychopathic Traits in Sporting Environments
Source: Sports (Basel). 2023 Nov 15;11(11):228. doi: 10.3390/sports11110228 (PMC10674364; doi:10.3390/sports11110228)
Supplement: Supplementary file 1 [file sports-11-00228-s001.zip › sports-2657225-supplementary.pdf]

**Table S1**. Studies Discussing Psychopathy in Sporting Contexts

| Author | Year | Participants | Instrument |
|---|---|---|---|
| Stanger, N., Kavussanu, M., Willoughby, et al., [44] | 2012 | n=66 Medium-High Contact (Team) Sport Athletes<br><br>n=40 male<br>n=26 female | Moral Disengagement in Sport Scale–Short (MDS-S), Interpersonal Reactivity Index (IRI), Prosocial and Antisocial Behavior in Sport Scale (PABSS), Self-Report Psychopathy scale III (SRP-III), Self-Assessment Manikin, and other biodata |
| Nicholls, A.R., Madigan, D.J., Backhouse, et al [45] | 2017 | n=285 Competitive athletes<br><br>n= 217 male<br>n=68 female | A study used the 8-item version of the Performance Enhancement Attitude Scale (PEAS) and the Short Dark Triad (SD3) |
| Ueno, Y., Shimotsukasa, T., Suyama, S., et al [33] | 2017 | n=506 Athletes from 42 different disciplines<br><br>n=217 male<br>n=289 female | Demographic questions plus the Dark Triad Dirty Dozen (DTDD) |

| | | | |
|---|---|---|---|
| Vaughn, Carter, Cockroft, et al [46] | 2018 | n=762<br>Team and individual sport athletes<br><br>n=355 male<br>n=407 female | Mental Toughness Questionnaire-48, SD3, and The International Physical Activity Questionnaire Short Form (IPAQ) |
| Nogueira, A., Tovar-Gálvez, M., González-Hernández, J., [47] | 2019 | n=241 Amateur endurance athletes<br><br>n=148<br>n=93 female | A study used Exercise Addiction Inventory (EAI), Grit-S, and SD3 |
| Vaughan, R., Madigan, D.J., Carter, G.L., et al [48] | 2019 | n=1258<br>Team and individual sport athletes<br><br>n=633 male<br>n=625 female | SD3 |
| González-Hernández, J., Cuevas-Campos, R., Tovar-Gálvez, et al[49] | 2020 | n=806<br>Professional and amateur athletes, varying disciplines<br><br>n=483 male<br>n=323 female | SD3 and the Competitiveness Scale-10 (C-10). |

| | | | |
|---|---|---|---|
| Nicholls, A.R., Madigan, D.J., Duncan, L., et al [50] | 2020 | n=164<br>Team and individual sport athletes<br><br>n=95 male<br>n=69 female | SD3, Short-Form Performance Enhancement Attitude Scale (SF-PEAS), and a number matrix task. |
| Vaughan, R.S., Madigan, D.J, [31] | 2020 | n=189<br>Basketball players<br><br>n=110 male<br>n=79 female | SD3, Multidimensional Competitive Orientation Inventory (MCOI), and basketball free throws. |
| González-Hernández J., Baños R., Morquecho-Sánchez R et al [51] | 2021 | n=426<br>Crossfit users and runners<br><br>n=257 male<br>n=169 female | Multidimensional Perfectionism Scale (FMPS), SD3, and EAI |
| Greitemeyer, T. [52] | 2022 | n=811<br>Athletes from 46 different disciplines<br><br>n=291<br>n=520 female | Dark tetrad questionnaire, SDT, Assessment of Sadistic Personality questionnaire (ASP). |

| | | | |
|---|---|---|---|
| Sordia, N., Jauk, E., Martskvishvili, K [53] | 2022 | n=364<br>College students, including athletes<br><br>n= 162 male<br>n= 202 female | Inventory of Creative Activities and Achievements (ICAA), SDT, Big Five Inventory (BFI), Big Six Personality Dimensions (HEXACO), Narcissistic Personality Inventory (NPI), Hypersensitive Narcissism Scale (HSNS), Levenson Self-Report Psychopathy Scale (LSRP), and the Triarchic Psychopathy Measure (TRIPM). |
| Zamani Sani SH, Greco G, Fathirezaie Z, et al. [54] | 2023 | n =464 athletes (team and individual sports)<br><br>n=199 male<br>n=265 female | Dark Triad Scale (DTS), Insomnia Severity Index (ISI), Perceived Stress Scale (PSS), and the Moral Content Judgement in Sport Questionnaire (MCJSQ). |
| Piacentino D, Sani G, Kotzalidis et al [55] | 2022 | n=122<br>Gym athletes, bodybuilders<br><br>n=84 male<br>n=38 female | Self Report Sociodemographic Questionnaire, Structured Clinical Interview for DSM IV Axis I Disorders (SCID-I), Structured Clinical Interview for DSM IV Axis II Disorders(SCID-II), Hamilton Depression Rating Scale (HDRS), Hamilton Anxiety Rating Scale (HARS), Young Mania Rating Scale (YMRS), Hypomania Checklist-32 (HCL-32), Mood Disorder Questionnaire (MDQ), Sick, Control, One, Fat, Food Eating Disorder Screening Test (SCOFF), and Barratt Impulsiveness Scale(BIS-II). |

| | | | |
|---|---|---|---|
| Hauger LE, Havnes IA, Jørstad ML, Bjørnebekk A., [56] | 2021 | n=139 Weightlifters, gym athletes<br><br>n=139 male | Alcohol Use Disorders Identification Test (AUDIT), Drug Use Disorders Identification Test (DUDIT), Wechsler Abbreviated Scale of Intelligence (WASI), Buss Perry Aggression Questionnaire (BPAQ), Behavior Rating Inventory of Executive Function-Adult version (BRIEF-A), and the antisocial personality subscale from The Millon Clinical Multiaxial Inventory-III (MCMI-III). |
| Nelson Brian S, Hildebrandt Tom, Wallisch Pascal [57] | 2022 | n=492 gym athletes<br><br>n=492 males | Demographic/self-report on events, training, etc, Barratt Impulsiveness Scale (BIS-11), Levinson Self-Report Psychopathy Scale (LSRP), Generalized Anxiety Disorder 7-item scale, Center for Epidemiologic Studies Short Depression Scale (CES-D 10), State Hostility Scale |
| Bryan, W., Donachie, T.C., Vaughan, R.S., et al [58] | 2023 | n=420 Team and individual sport athletes + coach pairs<br><br>n=130 male<br>n=290 female | SD3 and the Competitive Aggressiveness, Anger Scale (CAAS) |

| | | | |
|---|---|---|---|
| Cook G.M., Fletcher D., Peyrebrune M [59] | 2021 | n=36 Swimming coaches<br><br>n=33 male<br>n=3 female | BFI, DTDD, and Schutte Emotional Intelligence Scale (SEIS). |
| Cook G.M., Fletcher D., Peyrebrune M [60] | 2022 | n=38 Olympic swimmers<br><br>n=18 male<br>n=20 female | BFI, DTDD, SEIS. For DTDD |
| Russell, G.W., [61] | 1995 | n=395 Hockey spectators<br><br>n=57 male<br>n=46 female | LSRP, assault subscale of the Buss Durkee Hostility Inventory (BDHI), Bown's self-esteem measure, anomy scale from McClosky and Schaar, and demographic data. |
| Russell, G.W., Arms, R.L.[62] | 1998 | n=78 Hockey spectators<br><br>n=78 male | Sub scales of the AQ, LSRP, Grush Impulsivity Scale (GIS) Thrill and Adventure Seeking and Boredom Susceptibility subscales of Zuckerman’s Sensation Seeking Scale (ZSSS), and Buss Scale of Public Self-consciousness. |

| | | | |
|---|---|---|---|
| Russell, G.W., Arms, R.L., Mustonen, A, [63] | 1999 | n=74<br>General sport spectators<br><br>n=74 male | Physical Aggression and Anger subscales of the Aggression Questionnaire (AQ), LSRP, GIS, Thrill and Adventure Seeking and Boredom Susceptibility sub scales of ZSSS, and Law and Order scale, and other biodata |
| Yoder KJ, Porges EC, Decety J. [64] | 2015 | n=43<br>MMA spectators<br><br>n=43 male | The Psychopathic Personality Inventory-Revised (PPI-R) and a brain scan (Philips Achieva 3T) |
| Međedović J, Kovačević U [65] | 2020 | n=246 football fans<br><br>n=246 males | Club Supporting Scale, Dirty Dozen questionnaire, Direct Sadism Scale, Measures of Criminal Attitudes and Associates, part B |
